# ‘Language has been granted too much power’.^1,p.1^ Challenging the power of words with time and flexibility in the precommencement stage of research involving those with cognitive impairment

**DOI:** 10.1111/hex.13576

**Published:** 2022-09-12

**Authors:** Louise Locock, Deirdre O'Donnell, Sarah Donnelly, Liz Ellis, Thilo Kroll, Éidín Ní Shé, Sara Ryan

**Affiliations:** ^1^ Health Services Research Unit University of Aberdeen Aberdeen Scotland; ^2^ School of Nursing, Midwifery and Health Systems University College Dublin Dublin Ireland; ^3^ UCD Centre for Interdisciplinary Research, Education and Innovation in Health Systems (IRIS) University College Dublin Dublin Ireland; ^4^ School of Social Policy, Social Work and Social Justice University College Dublin Dublin Ireland; ^5^ Institute of Health Research and Innovation University of the Highlands and Islands Inverness Scotland; ^6^ Graduate School of Healthcare Management Royal College of Surgeons in Ireland Dublin Ireland; ^7^ Department of Social Care and Social Work Manchester Metropolitan University Manchester UK

**Keywords:** cognitive impairment, inclusion, public and patient involvement, research culture, seldom heard

## Abstract

Meaningful and inclusive involvement of all people affected by research in the design, management and dissemination of that research requires skills, time, flexibility and resources. There continue to be research practices that create implicit and explicit exclusion of some members of the public who may be ‘seldom heard’ or ‘frequently ignored’. Our focus is particularly on the involvement of people living with cognitive impairment, including people with one of the many forms of dementia and people with learning disabilities. We reflect especially on issues relating to the precommencement stage of research. We suggest that despite pockets of creative good practice, research culture remains a distinct habitus that continues to privilege cognition and articulacy in numerous ways. We argue that in perpetuating this system, some researchers and the institutions that govern research are committing a form of bureaucratic violence. We call for a reimagining of the models of research governance, funding and processes to incorporate the time and flexibility that are essential for meaningful involved research, particularly at the precommencement stage. Only then will academic health and social science research that is truly collaborative, engaged, accessible and inclusive be commonplace.

**Public and Patient Contribution:**

This viewpoint article was written by a research network of academics with substantial experience in undertaking and researching patient and public involvement and codesign work with representatives of the public and patients right across the health system. Our work guided the focus of this viewpoint as we reflected on our experiences.

## 1 BACKGROUND

Research funders and universities, supported by government policy, continue to promote the involvement of people impacted by research in the design, management, and dissemination of that research. In health research, this may include patients living with a particular condition, their family members, carers (paid and unpaid) and members of the public.

This viewpoint summarizes the discussions of a network of researchers from Ireland, Scotland and England with a track record in undertaking coresearch/design and researching patient and public involvement (PPI), particularly with people living with cognitive impairment, including people with one of the many forms of dementia and people with learning disabilities. This network was funded as part of a joint programme between the UK Economic and Social Research Council and the Irish Research Council. This grant aimed at fostering social science networks between the United Kingdom and Ireland.

We acknowledge that terms and labels can be contentious and are not universally agreed upon and accepted. Ongoing debates on the history and use of language around disability continue to highlight the power of language to perpetuate stigma and discrimination.^1^, ^2^, ^3^, ^4^ We use the term ‘cognitive impairment’ to mean anyone who experiences issues in terms of memory, reading, comprehension or thinking. The focus of our conversations was on the precommencement stage of research. This stage ‘Includes the time before a research project/partnership starts or when funding is being applied for.’^5^ ^,p.1^ Understanding, building trust, and engaging with partners are key activities at the precommencement stage. From our experiences and reflections, this is a point where inequalities of power and agenda‐setting have already started to shape and constrain how the research evolves. For all public involvement, but particularly for involving people with cognitive impairment, the time, skills and resources needed to make meaningful and genuine partnerships are often lacking, which leads to unintended consequences before research projects commence.^6^, ^7^

## 2 ‘SELDOM HEARD’—WHAT DOES THIS REALLY MEAN?

It is important to unpack what the term ‘seldom heard’ means for academic research. We recognise that the term itself is an acknowledgement that some groups of people may be marginalized by research practice when they are seen as a uniform group through the label of ‘their condition’. However, this term is problematic because it shifts the emphasis away from the researcher to the social group in question. People described as ‘seldom heard’ often share an overarching condition that subsumes their individual personalities, abilities, social group memberships, and other identities, such as a parent, sister, artist or musician. Differences in personality characteristics, general diversity, and intersectionality are often neglected. For example, more introverted people or those who do not feel comfortable with sharing their experiences in group settings may be overlooked. People living in precarious circumstances or in remote locations may not be approached for research projects or those who belong to otherwise socially marginalized population groups, like refugees, migrants or homeless people may not be actively involved in research.

The second aspect in relation to ‘seldom heard’ is social marginalization or exclusion due to perceived limitations of people's cognitive capacity that risks positioning and misrepresenting them as unable to contribute. This form of exclusion may be accepted and even embedded into research practice.^8^ This means that people are often not involved in research as they are not seen as ‘fit’ or competent, or even too ‘vulnerable’ to be approached and engaged. Thus, they become largely invisible and research about them without their voice and representation is perpetuated.

‘Seldom heard’ is also a product of how the communication process itself is created. Usually, researchers who are in a position of power and privilege due to their academic status develop the initial approach. Responding to time and budget pressures, and their inherent preferences for how communication needs to unfold means there may be a tendency to accept those who are the first to respond to the study documentation and who may be the ‘easiest’ to engage with. Typically, this means educated, neurotypical, verbal and confident individuals. So, people with cognitive impairments and other ‘seldom heard’ groups may be overlooked and excluded through recruitment strategies and approaches. It is suggested that so‐labelled people are not so much ‘seldom heard’ as seldom listened to, easily ignored or not even thought about.^9^

Under legislation in all jurisdictions within the network, capacity must be assumed until proven otherwise.^10^, ^11^, ^12^ Therefore, the onus is on us as researchers to provide the necessary ‘scaffolding’ to facilitate participation. However, often, the communication arrangements (i.e., assistance, alternative formats) may not be appropriate or tailored to individual circumstances to allow for a meaningful engagement. Underpinning these processes is the need for additional time, flexibility and relationship building. Reflecting on our own experiences of developing research partnerships and codesign teams with people who have cognitive impairments we note that time, in particular, must be recognized as a significant resource requirement for authentic participation. Yet from our experience, time is often the most constrained of resources, particularly in precommencement stages when working without a budget towards funder deadlines.

## 3 BUREAUCRATIC VIOLENCE AND ‘ETHICAL LONELINESS’

A collective research system that continues to privilege cognition and articulacy is arguably committing what might be termed institutional or bureaucratic violence against those less able to take part in such a verbal culture. Galtung^13^ suggested that violence was not simply an interpersonal act of force but could also arise from more anonymous institutional structures and systems. These create a monopoly of power and promote the interests of one group of people over another group, who are then unable to realize their full capabilities. But these structures are impersonal and silent, without a single identifiable ‘perpetrator’.

In their book ‘Ethical Loneliness: The Injustice of Not Being Heard’ Stauffer^14^ ^,pp.1–2^ describes ethical loneliness as ‘The experience of having been abandoned by humanity compounded by the experience of not being heard….a form of social abandonment that can be imposed only by multiple ethical lapses’ on the part of human beings and political institutions. Crucially Stauffer^14^ ^,p.2^ argues it is not simply a result of deliberate oppression or dehumanization but also often ‘By the failure of just‐minded people to hear well’.

Arguably researchers do not consciously want to exclude people with cognitive impairment from research processes, indeed many researchers are doing their utmost as individuals to include them. However, working within a system of implicit exclusion whereby alternative forms of involvement do not fit prevailing norms requires researchers to be doggedly persistent in pursuing meaningful inclusion.^15^

## 4 WHAT ARE WORDS WORTH? THE WRITTEN AND SPOKEN CULTURE OF RESEARCH

At the heart of this issue is the written and spoken culture of research. Research and the preparation of research grant applications is an inherently wordy process. To meet funding requirements, lengthy and often quite technical forms need to be prepared. This poses potential challenges for many people who are unfamiliar with research practices; the difficulties of impenetrable, technical jargon and burdensome paperwork are well documented.^16^ Lay summaries may make the content of research applications and protocols more accessible to a wider audience, but do not replace the need for written detail. These wordy research practices can be doubly exclusionary for people who experience issues around memory, reading, comprehension or thinking. As Barad,^1^ ^,p.1^ quoted in the title for this viewpoint, argues ‘Language has been granted too much power’. This form of power perpetuates the further marginalization of those voices it is important to hear. This might be considered a form of epistemic injustice, whereby the ways of communicating knowledge and experience by one group of people are rendered inadmissible or ineligible by the practices of another.^17^, ^18^

In a recent study of PPI using an epistemic justice framework, Liabo et al.^19^ draw on Fricker's^17^ concepts of testimonial and hermeneutical injustice. In testimonial injustice, the person's experiential account is not taken seriously because they are not a researcher or practitioner. Particularly relevant for this paper is hermeneutical injustice, ‘when public collaborators do not have the conceptual tools to interpret their experiences of healthcare or to contribute their experiential knowledge’.^19^ ^,p.2^ Liabo et al.^19^ ^, p.10^ note the need for ‘a shared syntax to represent knowledge’ and describe sometimes considerable work undertaken by public collaborators to ‘turn their narratives into “useful” contributions to a scientific discussion’.

These practices that privilege cognition and articulacy also risk excluding researchers themselves from a meaningful partnership, limiting their access and preventing them from ‘hearing’ or understanding the perspectives, language and lifeworld of people with cognitive impairments. Even when establishing this academic network, we were constrained by funder systems from including public and patient coapplicants. Therefore, in our collaborations and in drafting this paper, we have been conscious of the paradox that in the very act of seeking to articulate the issue we may be perpetuating boundaries and reinforcing difference.

Despite pockets of creative good practice in dismantling these boundaries,^20^ research culture thus remains a distinct habitus that continues to privilege cognition and articulacy in numerous ways. For example, a network member had a recent experience of responding to a lay reviewer for a grant proposal who argued that the lay summary of the proposal was too simple and requested more technical explanations of the proposed methodology. This was despite the lay summary being written by public and patient coapplicants for an audience that may include people with cognitive impairment. Even the words we use around whether people are ‘given a voice’, or are seldom ‘heard’, imply an ability to ‘tell’—to understand what questions researchers are asking, to articulate an idea, to remember and order relevant aspects of one's experience. Taking part in a James Lind Alliance Priority Setting Partnership to identify research priorities, for example, requires survey responses or taking part in a meeting. Becoming a peer researcher or coapplicant on a grant can require people to negotiate obstructive online funding application forms, create online accounts, read and sign contracts or confidentiality agreements, fill in expenses or payment forms or sign to say they meet the criteria for coauthorship. Like many public and private organizations, universities, academic journals and research funding and approval systems have an uncanny ability to think of the worst possible bit of bureaucracy and double it.

Of course, we need to be wary of patronising assumptions that no one with a cognitive impairment can work with these systems. Some people may have no difficulty or may be able to manage well, either independently or with appropriate support from a family member or support worker or research project manager. However, we suggest that this may create a selection bias towards partners who can negotiate our ‘wordy world’ and by extension exclude those who cannot from being peer researchers, coapplicants or coauthors. One network member told us of a meeting with people with learning disabilities at which one person objected strongly to the use of pictograms, saying ‘We're not stupid, we can read, you know?’, but was countered by another person saying ‘Well I can't read, so I like the smiley faces’. Another described with some despair the ‘layers of barriers’ and protracted process of getting the university to set up accessible freelance contracts for people with learning disabilities to join researchers as ethnographic observers. There were challenges in relation to indemnity insurance as well as overly complex processes for submitting invoices to be paid.

## 5 SOLUTIONS: TIME, FLEXIBILITY AND MUTUAL VULNERABILITY

A genuine partnership with people with cognitive impairment may look and feel very different from what we traditionally understand as PPI. While terms such as coproduction are increasingly invoked in PPI processes, actual practices are often still far removed from what a participatory action researcher might see as research designed and done together for the benefit of the community served. With reference to the precommencement phase, there remain remarkably few examples of research ideas genuinely instigated and designed by people with cognitive impairment. A person with lived experience of cognitive impairment and who has been involved in academic research said to us, ‘We want to be there at the planning stage, not when they're already laying the foundations of the building’.

Funders, while advocating for PPI and coproduced approaches,^21^ often going so far as to make it a condition of funding, seldom support this study in the precommencement stage. This raises significant ethical issues around working with the public in ways that do not exploit the goodwill and labour of marginalized groups. Researchers who are seeking to involve people living with cognitive impairment often encounter specific challenges when making the case to funders for adequate PPI budgetary provision. This includes a provision to support the engagement of those who may have particular resource requirements, such as alternative communication technology, decision‐supporters or personal assistants. Researchers who specialize in research about different forms of cognitive impairment may have painstakingly found ways around structural and process barriers but at some personal and career cost.

To conclude building relationships, using flexible creative methods and finding ways to incorporate the experiences of those who, metaphorically or literally, have no ‘voice’ at all, is not a one–off interaction. It requires time, patience, skill and often money—and yet pregrant work‐up is frequently done in a rush and on a shoestring. Meanwhile, the participation of people with cognitive impairment in designing other research that may affect them, for example, trials on heart disease, hip fracture or epilepsy, remains a rarity. This is reflected in the failure to include people with cognitive impairment, even as participants, in much research into physical health.^8^, ^22^ The National Institute for Health and Care Research Include Framework is rightly raising the need to be more inclusive in recruiting trial participants, but the same needs to apply to prestudy design.^23^

We recognize that for any researcher, but particularly for those who do not routinely work on topics relating to types of dementia or learning disabilities, the challenge to ‘get it right’ can feel daunting, and there is potential for hurt and harm to ensue, both for the people they seek (or fail) to involve and inexperienced and experienced researchers. We suggest that established and formalized communities of practice could be a source of advice and support to researchers. These communities of practice might bring together experienced researchers, with public and patient stakeholders and collate examples of good practice for researchers who wish to collaborate with those who are seldom heard and who are negotiating the demands of funders and ethics committees. These communities of practice might also coproduce training courses and materials around involvement and support the democratizing of research processes by challenging the infrastructural and bureaucratic barriers to meaningful participation. This necessitates a reimagining of current models of research governance and processes to incorporate the time and flexibility that are essential for involved research, particularly at the precommencement stage. Only then will academic health and social science research that is truly collaborative, engaged, accessible and inclusive become commonplace.

## AUTHOR CONTRIBUTIONS

All the authors have made significant intellectual and practical contributions to this viewpoint. Louise Locock wrote the original manuscript. All authors reviewed a draft and inputted feedback or edits. Deirdre O'Donnell prepared the final submission. Louise Locock and Deirdre O'Donnell are the principal investigators for the project and the grant holders. All authors have read and agreed to the published version of the manuscript.

## CONFLICT OF INTEREST

The authors declare no conflict of interest.

## Data Availability

Data sharing is not applicable to this article as no datasets were generated or analysed during the current study.

## References

[1] Barad K . Posthumanist performativity: toward an understanding of how matter comes to matter. Signs. 2003;28(3):801‐831. 10.1086/345321

[2] Shakespeare T . Disability Rights Wrongs Revisited. 2nd ed. Routledge; 2014.

[3] Sabat SR . The Experience of Alzheimer's Disease: Life through a Tangled Veil. Wiley‐Blackwell; 2001.

[4] Swaffer K . Dementia: stigma, language, and dementia‐friendly. Dementia. 2014;13(6):709‐716. 10.1177/1471301214548143 25326220

[5] Ní Shé É , Cassidy J , Davies C , et al. Minding the gap: identifying values to enable public and patient involvement at the pre‐commencement stage of research projects. Res Involv Engagem. 2020;6(1):46. 10.1186/s40900-020-00220-7 32765898 PMC7396939

[6] Ní Shé É , Harrison R . Mitigating unintended consequences of co‐design in health care. Health Expect. 2021;24(5):1551‐1556. 10.1111/hex.13308 34339528 PMC8483209

[7] Mulvale G , Moll S , Miatello A , et al. Codesigning health and other public services with vulnerable and disadvantaged populations: insights from an international collaboration. Health Expect. 2019;22(3):284‐297. 10.1111/hex.12864 30604580 PMC6543156

[8] Spaul SW , Hudson R , Harvey C , Macdonald H , Perez J . Exclusion criterion: learning disability. Lancet. 2020;395(10223):e29. 32061300 10.1016/S0140-6736(20)30051-9

[9] Escobar O , Morton S , Lightbody R , Seditas K . Hard to reach or easy to ignore: promoting equality in community engagement‐evidence review. 2017. Accessed August 4, 2022. http://whatworksscotland.ac.uk/publications/hard-to-reach-or-easy-to-ignore-promoting-equality-in-community-engagement-evidence-review/

[10] legislation.gov.uk . Adults with Incapacity (Scotland) Act 2000 [Internet]. Accessed August 4, 2022. https://www.legislation.gov.uk/asp/2000/4/contents

[11] legislation.gov.uk . Mental Capacity (England and Wales) Act 2005 [Internet]. Sect. 1(2). Accessed August 4, 2022. https://www.legislation.gov.uk/ukpga/2005/9/contents

[12] Houses of the Oireachtas . Assisted Decision Making Capacity Act 2015 (Ireland) [Internet]. Accessed August 4, 2022. https://www.oireachtas.ie/en/bills/bill/2013/83/

[13] Galtung J . Violence, peace, and peace research. J Peace Res. 1969;6(3):167‐191. 10.1177/002234336900600301

[14] Stauffer J . Ethical Loneliness: The Injustice of not being Heard. Columbia University Press; 2015.

[15] Jahagirdar D , Kroll T , Ritchie K , Wyke S . Patient‐reported outcome measures for chronic obstructive pulmonary disease. Patient. 2013;6(1):11‐21. 10.1007/s40271-013-0004-5 23417577 PMC3585908

[16] Ocloo J , Matthews R . From tokenism to empowerment: progressing patient and public involvement in healthcare improvement. BMJ Qual Saf. 2016;25(8):626‐632. 10.1136/bmjqs-2015-004839 PMC4975844 26993640

[17] Fricker M . Epistemic Injustice: Power and the Ethics of Knowing. Oxford University Press; 2007.

[18] Hutchison K , Rogers W , Entwistle VA . Addressing deficits and injustices: the potential epistemic contributions of patients to research. Health Care Anal. 2017;25(4):386‐403. 10.1007/s10728-016-0323-5 27277736

[19] Liabo K , Cockcroft EJ , Boddy K , Farmer L , Bortoli S , Britten N . Epistemic justice in public involvement and engagement: creating conditions for impact. Health Expect. 2022;25(4):1967‐1978. 10.1111/hex.13553 35774005 PMC9327822

[20] Burton A , Ogden M , Cooper C . Planning and enabling meaningful patient and public involvement in dementia research. Curr Opin Psychiatry. 2019;32(6):557‐562. 10.1097/YCO.0000000000000548 31306247

[21] Public Involvement Standards Development Partnership . UK Standards for Public Involvement: Better public involvement for better health and social care research [Internet]. UK: National Institute for Health Research, Chief Scientist Office, Health and Care Research Wales, Public Health Agency; 2017. Accessed August 4, 2022. https://sites.google.com/nihr.ac.uk/pi-standards/standards?authuser=0

[22] Shepherd V . Advances and challenges in conducting ethical trials involving populations lacking capacity to consent: a decade in review. Contemp Clin Trials. 2020;95:106054. 32526281 10.1016/j.cct.2020.106054 PMC7832147

[23] Treweek S , Banister K , Bower P , et al. Developing the INCLUDE ethnicity framework—a tool to help trialists design trials that better reflect the communities they serve. Trials. 2021;22(1):337. 10.1186/s13063-021-05276-8 33971916 PMC8108025

